# Challenges in Diagnosis of COVID-19 Pneumonia under Ocrelizumab and De-Risking Strategies in Multiple Sclerosis—The Elephant Is (Still) in the Room

**DOI:** 10.3390/microorganisms12101941

**Published:** 2024-09-25

**Authors:** Alice Mariottini, Antonio Lotti, Valentina Damato, Luca Massacesi

**Affiliations:** 1 Department of Neurosciences, Psychology, Drug Research and Child Health (NEUROFARBA), University of Florence, 50139 Florence, Italy; 2 Neurology II Unit, Careggi University Hospital, 50134 Florence, Italy

**Keywords:** COVID-19, SARS-CoV-2, pneumonia, monoclonal antibody, anti-CD20, B cell depletion, ocrelizumab, multiple sclerosis, bronchoalveolar lavage, nasopharyngeal swab

## Abstract

Severe SARS-CoV-2 infections may still be observed in people bearing risk factors, such as the use of anti-CD20 monoclonal antibodies (mAbs), which are adopted in several autoimmune disorders including multiple sclerosis (MS). COVID-19 diagnosis is routinely based on nasopharyngeal swab testing, but suboptimal sensitivity for SARS-CoV-2 detection compared to bronchoalveolar lavage (BAL) may lead to misdiagnosis in some cases. Such diagnostic issues were described in a few MS patients receiving anti-CD20 mAbs, including middle-aged people and lacking information on subsequent MS therapeutic management, a debated topic as no evidence-based guidance on de-risking strategies is currently available. Here, we report the case of a young MS patient who developed severe COVID-19 pneumonia under treatment with the anti-CD20 mAb ocrelizumab, and who was finally diagnosed with SARS-CoV-2 by BAL despite repeatedly negative nasopharyngeal swabs. Ocrelizumab was then discontinued, and treatment with a sphingosine-1 phosphate receptor modulator was started, followed by maintenance of clinical and radiological MS stability. Challenges in diagnosing COVID-19 pneumonia in people without risk factors other than immunomodulatory treatment are hence discussed, as well as potential strategies for de-risking MS therapies. The latter topic is increasingly debated based on raising concerns for potential long-term safety issues of high-efficacy treatments, including anti-CD20 mAbs.

## 1. Introduction

At the time of the coronavirus 19 disease (COVID-19) outbreak, severe concerns for potentially life-threatening infections led to the worldwide adoption of containment measures and vaccination campaigns [1]. Four years later, anti-SARS-CoV-2 surveillance has declined and restrictions have been eased across countries [2]. Nonetheless, severe COVID-19 infections are still observed, especially in individuals bearing risk factors, such as older age, comorbidities, and definite types of immune suppression [3,4]; amongst these is the use of anti-CD20 monoclonal antibodies (mAbs), which are adopted in several autoimmune disorders including multiple sclerosis (MS) [5].

Nasopharyngeal swabs are the mainstay for COVID-19 diagnosis, but negative testing should not rule out COVID-19 in patients with high clinical suspicion, as bronchoalveolar lavage (BAL) can detect SARS-CoV-2 infection in a not-negligible proportion of such cases [6,7]. However, BAL is a minimally invasive procedure requiring a specific setting and expertise; therefore, it is not routinely performed in all cases of suspected pneumonia, possibly leading to misdiagnosis and suboptimal therapeutic management.

To our knowledge, few cases of COVID-19 pneumonia diagnosed by BAL after negative nasopharyngeal swabs in MS patients receiving anti-CD20 mAbs are reported in the literature [8,9]. All these include middle-aged patients, and often pneumonia was described in the context of prolonged viral shedding, which is known to be associated with anti-CD20 therapy [10]. However, information regarding subsequent therapeutic management is lacking, a challenging issue as no evidence-based guidance on de-risking strategies in MS is available so far.

Here, we report the case of a young MS patient treated with the anti-CD20 mAb ocrelizumab, who was diagnosed with COVID-19 pneumonia by BAL after being misdiagnosed with bacterial pneumonia due to repeatedly negative nasopharyngeal swabs; de-risking therapeutic strategies after ocrelizumab discontinuation are also discussed.

## 2. Case Presentation

A 35-year-old man was diagnosed with relapsing–remitting (RR-) MS at age 22, presenting with right-sided retrobulbar optic neuritis, with evidence of demyelinating lesions on brain magnetic resonance imaging (MRI) and oligoclonal bands in the cerebrospinal fluid. His past medical history was unremarkable. He was treated with interferon beta and dimethyl-fumarate for 9 and 1 years, respectively, both discontinued due to MS clinical and radiological activity (relapse and new MRI lesions). He was then started on ocrelizumab treatment, followed by clinical and radiological stabilization, with no residual disability (expanded disability status scale [EDSS] score: 1.0). He received the sixth dose of ocrelizumab in August 2023. White blood cell count tested one month later showed a Common Terminology Criteria for Adverse Events (CTCAE) [11] grade III neutropenia (520 cells/mm^3^) with spontaneous resolution after one week (2520 cells/mm^3^).

**Figure 1:**
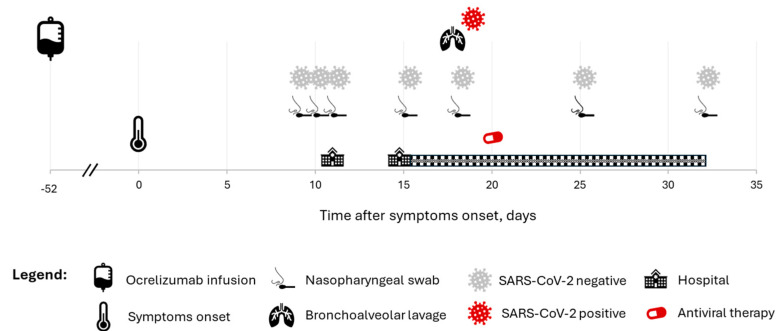
Timeline of nasopharyngeal swab testing and bronchoalveolar lavage (BAL) with respect to fever onset (day 0). Overall, 7 nasopharyngeal swabs (2 taken at home, and 5 at the hospital) tested negative for SARS-CoV-2, including 1 performed the same day of BAL, which tested positive. The 2 latest nasopharyngeal swabs were performed after antiviral therapy started.

In September 2023, seven weeks after the last dose of ocrelizumab, he developed fever associated with a headache and cough. At that time, according to local regulations, he had already received a full primary vaccination course with anti-SARS-CoV-2 mRNA vaccines (III doses, latest in winter 2022). Two nasopharyngeal swabs performed at home tested negative for COVID-19 (Figure 1). He was then started on antimicrobial therapy and low-dose corticosteroids for suspicion of sinusitis, which was incidentally observed in a routine brain MRI scan performed in that period. Blood tests taken a few days later showed mild leukopenia (white blood cells 3060/mmc, neutrophils 1930/mmc, lymphocytes 770/mmc) and a slight elevation in C-reactive protein (CRP; 1.69 mg/dL, upper limit of normal [ULN] 0.5) and erythrocyte sedimentation rate (ESR; 34, ULN 20); IgMs were moderately reduced (26 mg/dL, lower limit of normal [LLN] 40), being IgG and IgA within normal limits (1040 and 442 mg/dL, respectively). Due to the persistence of fever, one week later, he was admitted to the emergency department (ED) where he had a third nasopharyngeal swab, which was again negative for SARS-CoV-2. A chest X-ray showed parenchymal consolidation in the left lung, and BAL was positive for H. Influenzae; SARS-CoV-2 was not tested in that instance. He was then diagnosed with H. Influenzae pneumonia and discharged with ceftriaxone therapy. However, three days later, he returned to the ED due to the persistence of fever and worsening of dyspnoea. A nasopharyngeal swab was repeated, testing negative again. He then received a chest computed tomography (CT) scan showing extensive lung involvement with a crazy-paving pattern. Due to the persistence of fever and dyspnoea requiring low-flow oxygen therapy, a second BAL was performed which was positive for SARS-CoV-2 RNA; notably, a further nasopharyngeal swab, which was taken one hour before the BAL, tested negative. The patient was then treated with antiviral therapy (Nirmatrelvir/Ritonavir and Remdesivir) followed by resolution of fever and progressive clinical improvement. Three weeks after hospital admission, he was discharged with the diagnosis of “Severe COVID-19 pneumonia with superimposed H. Influenzae infection”, and a few months were required to achieve a complete clinical recovery. He experienced COVID-19 reinfection in January 2024, which was promptly treated with antiviral therapy with clinical resolution.

After discussing with the patient potential options for MS therapeutic management, ocrelizumab was discontinued due to a perceived unfavourable risk–benefit ratio. Given the minimal disability and absence of MS activity over the past 4 years, in April 2024 (i.e., 8 months after the last dose of ocrelizumab), the patient was switched as a de-risking strategy to the sphingosine 1-phosphate receptor modulator (i-mod) ponesimod. The lymphocyte count dropped from the first month after treatment commencement, stabilizing as a grade II CTCAE lymphopenia [11]. The patient is still receiving ponesimod at the time of writing of this report (September 2024); no new MS symptoms nor any other complications were observed so far, and a brain MRI performed in August 2024 was stable compared to the previous examination from September 2023.

## 3. Discussion

In this report, we describe the case of a man in his thirties affected by RR-MS without disability or comorbidities receiving ocrelizumab treatment, who experienced severe COVID-19 pneumonia which was at first misdiagnosed as H. Influenzae pneumonia due to repeated nasopharyngeal swabs testing negative for SARS-CoV-2.

A potentially increased risk for severe COVID-19 has been suggested to be associated with the use of disease-modifying treatments (DMTs) for MS, especially anti-CD20 mAbs [5], which are associated with a reduced anti-SARS-CoV-2 humoral response to both vaccination and infection compared to other DMTs, but similar or even increased cell-mediated response [12,13,14]. Notwithstanding, severe infections were described only in a minor proportion of patients, being usually associated with older age, higher disability, progressive phenotype, comorbidities, and rituximab use [15,16]. Such events were estimated to be even rarer after anti-SARS-CoV-2 vaccination [17]. Based on these findings, the low a priori risk for severe COVID-19 along with repeatedly negative nasopharyngeal swabs likely contributed to the initial misdiagnosis in our patient, which could have in turn affected disease severity. However, the sensitivity of a nasopharyngeal assay is known to be lower compared with BAL, being estimated at roughly 89% in critically ill patients [18]. In cases bearing high clinical suspicion, COVID-19 should hence not be ruled out based on a negative nasopharyngeal swab, but a comprehensive diagnostic work-up likely including BAL should be undertaken [19]. Compared to nucleic acid amplification tests, which are the mainstay for COVID-19 diagnosis, SARS-CoV-2 antigen tests show an overall specificity ≥ 99%, but low or moderate sensitivity dependent on the presence or absence of COVID-19 symptoms and the time of testing after symptom onset, ranging from 89% for symptomatic individuals tested within the first five days of illness to 54% after 5 days, being even lower in asymptomatic individuals [20]. Similarly, serology testing for anti-SARS-CoV-2 antibodies shows low sensitivity over the first two weeks after symptoms onset, and it is currently not recommended for the diagnosis of acute infection by the Infectious Diseases Society of America Guidelines [21]. Serology testing may be useful to provide evidence of previous SARS-CoV-2 infection when performed three to five weeks after symptom onset [21], but its sensitivity in immunocompromised patients may be hampered by reduced humoral response to the virus. Although similar diagnostic challenges were reported in few published cases of MS patients receiving anti-CD20 mAbs [8,9], to our knowledge, this has never been described in vaccinated young MS patients without any risk factors for severe infections, except for the immunomodulatory DMT. In our case, transient neutropenia and a slight reduction in IgM could represent additional risk factors for infective complications under ocrelizumab treatment, as recently described [22,23].

Besides COVID-19, other common infections may burden the use of immunomodulatory/suppressive treatments for MS and other autoimmune disorders. Guidelines on this topic are available, suggesting the adoption of protective strategies against infections, as well as a schedule of vaccination including varicella, measles, mumps, rubella, tetanus, hepatitis B, and other infections according to the local epidemiological context and based on the patient’s natural immunity, vaccine history, and the results of the pre-vaccine serologic tests [24]. Similar vaccination schedules are recommended for people with other autoimmune disorders, such as systemic lupus erythematosus or systemic sclerosis [25,26].

After an 8-month wash-out period, ponesimod was reintroduced as a de-risking strategy. Although no guidance is currently available on this topic, some hints can be inferred from studies on DMT discontinuation, where prolonged disease stability predicted a successful treatment withdrawal in patients aged more than 55–60 years [27,28]. De-risking with a moderate- to high-efficacy (HE-)DMT could therefore likely be safely offered to young patients with prolonged disease stabilization and safety issues under a definite HE-DMTs. Furthermore, the use of anti-CD20 mAbs as a potential “induction strategy” followed by de-escalation to oral DMTs was suggested, and the need for de-risking anti-CD20 therapies in the long term is increasingly debated [29].

The choice of the i-mod class was motivated by the lack of evidence for an increased risk of severe COVID-19 associated with its use [30]. Intriguingly, fingolimod was also explored as a potential treatment strategy for people with moderate to severe COVID-19 [31], and a protective effect against SARS-CoV-2-induced hyper-inflammation was suggested for this DMT class, based on both S1P receptor-dependent and -independent mechanisms [32,33]. Even if most COVID-19-related reports refer to fingolimod, to our knowledge, no data support a higher risk for severe COVID-19 in patients receiving ponesimod. Furthermore, available evidence suggests that the use of ponesimod and other newer-generation i-mods is associated with higher seroconversion rates compared to fingolimod, possibly offering a more effective infection control and anti-viral response to SARS-CoV-2 and other pathogens [34,35].

In this case, ponesimod was preferred over the remaining i-mod for the following reasons: (i) higher selectivity for S1P_1_ receptor subtype compared to fingolimod, not requiring first-dose electrocardiographic monitoring; (ii) the plausible lowest risk for pharmacological interactions due to disposal by multiple independent metabolic pathways [36,37], which could also be relevant in cases of concomitant administration of some anti-SARS-CoV-2 therapies [34]; (iii) it shows the most rapid kinetic of lymphocyte repopulation after withdrawal [38], which could allow short treatment breaks to improve vaccine responses without risks for MS rebound [34,39] and provide additional safety in case of future infective complications. For the latter reason, DMTs with long-lasting effects on the immune system like alemtuzumab and cladribine were excluded. Natalizumab was considered, but excluded after careful consideration of the patient’s convenience and the current favourable prognostic factors, including the complete suppression of inflammatory activity over the past 4 years and minimal residual disability with low lesion load. First-line DMTs were excluded as two DMTs of this class were previously failed by the patient.

Clinical and radiological MS activity was suppressed so far, without any further infective complications. Even if a longer follow-up is required to confirm the safety profile, it could be speculated that the risk could have been highest in the first months, based on the kinetics of B cell repopulation after ocrelizumab treatment. Similarly, longer follow-up is needed to ascertain whether ponesimod’s effectiveness is maintained over time.

Besides the short follow-up after the treatment switch, another limitation of this report is the lack of information on anti-SARS-CoV-2 antibody titres; however, reduced antibody-mediated response to SARS-CoV-2 under anti-CD20 therapy compared to other DMTs was previously reported [12]. In addition, as more than six months had passed between the latest vaccine dose and the index event, it cannot be excluded that vaccination efficacy had waned over time due to the circulation of novel SARS-CoV-2 variants and a potential drop in humoral responses. Aligned with this observation, we previously reported a 21% incidence rate of COVID-19 within 7 months from the booster dose [40]. On the other hand, a stable antibody reactivity was described up to 180 days after the booster dose [41], with a stronger effect for mRNA vaccines and a slower decline for people who received three vs. two doses of vaccine [42].

## 4. Conclusions

The present report contributes to drawing further attention to the risk for severe COVID-19 in MS patients without any clinical or demographical risk factors other than immunomodulatory treatment. In these cases, diagnostic suspicion of COVID-19 pneumonia should not be overlooked, and a comprehensive diagnostic work-up (likely including BAL) should be performed [19]. As, in such cases, the benefit/risk ratio of maintaining the same DMT could be deemed as unfavourable, further evidence on potential de-risking strategies is needed to assist the clinician in treatment decision making.

## Data Availability

The original contributions presented in the study are included in the article; further inquiries can be directed to the corresponding author.
